# Increasing Warmth in Adolescents with Anorexia Nervosa: A Randomized Controlled Crossover Trial Examining the Efficacy of Mustard and Ginger Footbaths

**DOI:** 10.1155/2020/2416582

**Published:** 2020-01-30

**Authors:** S. Kuderer, E. Helmert, H. Szöke, S. Joos, M. Kohl, J. Svaldi, F. Beissner, F. Andrasik, J. Vagedes

**Affiliations:** ^1^ARCIM Institute (Academic Research in Complementary and Integrative Medicine), Im Haberschlai 7, 70794 Filderstadt, Germany; ^2^University of Pécs, Department of CAM, Hunyadi út 4, 7621 Pécs, Hungary; ^3^University of Tuebingen, Institute for General Practice and Interprofessional Care, Geschwister-Scholl-Platz, 72074 Tuebingen, Germany; ^4^University Furtwangen, Institute of Precision Medicine, Jakob-Kienzle-Straße 17, 78054 VS-Schwenningen, Germany; ^5^University of Tuebingen, Department of Psychology, Geschwister-Scholl-Platz, 72074 Tuebingen, Germany; ^6^Hannover Medical School, Institute for Diagnostic and Interventional Neuroradiology, Carl-Neuberg-Straße 1, 30625 Hannover, Germany; ^7^University of Memphis, Department of Psychology, 400 Innovation Drive, Memphis, 38152 TN, USA; ^8^University of Tuebingen, Children's Hospital, Hoppe-Seyler-Straße 1, 72076 Tuebingen, Germany

## Abstract

**Objective:**

To analyze the thermogenic effects of footbaths with medicinal powders in adolescents with anorexia nervosa (AN) in comparison to healthy controls (HCs). *Intervention and Outcomes*. Forty-one female participants (21 AN, 20 HCs; 14.22 ± 1.54 years) received three footbaths—warm water and mustard (MU, Sinapis nigra), warm water and ginger (GI, Zingiber officinale), or warm water only (WA), in random order within a crossover design. Data were collected before (t1), immediately after foot immersion (maximum 20 minutes) (t2), and after 10 minutes subsequently (t3). Actual skin temperature (high resolution thermography) and perceived warmth (HeWEF questionnaire) were assessed at each time point for various body parts. The primary outcome measure was self-perceived warmth at the feet at t3. Secondary outcome measures were objective skin temperature and subjective warmth at the face, hands, and feet.

**Results:**

Perceived warmth at the feet at t3 was significantly higher after GI compared to WA (mean difference −1.02) and MU (−1.07), with no differences between those with AN and HC (−0.29). For the secondary outcome measures, a craniocaudal temperature gradient for the skin temperature (thermography) was noted at t1 for patients with AN and HC (AN with colder feet). The craniocaudal gradient for subjective warmth was only seen for patients with AN.

**Conclusion:**

Footbaths with ginger increased warmth perception at the feet longer than with mustard or warm water only for adolescents with AN as well as for HC. The impact of ginger footbaths on recovery of thermoregulatory disturbances in patients with AN repeated over extended periods merits further investigation.

## 1. Introduction

The clinical picture of anorexia nervosa (AN) is frequently characterized by hypothermia (with core temperatures below 36.1°C) [1, 2], cold blue extremities, and a permanent sensation of cold [1, 3, 4]. In addition to the thermal discomfort of cold extremities [5], cold feet can increase susceptibility to infections [6] and contribute to disturbances in sleep behavior [7, 8]. Patients with AN often react to these symptoms with behavioral adaptions including drinking warm liquids and/or wrapping oneself in warming blankets, hot water bottles, and extralayers of clothing [1, 9]. Hints within the literature suggest that such strategies for increasing body warmth in individuals with AN do have subjective and symptomatic benefits [9, 10], as well as beneficial effects on recovery [11]. Hence, warming in individuals with AN by an external heat supply could optimize the complex treatment process [3]. Footbaths are frequently used to improve body warmth regulation [12]. Because of the high number of capillaries in the feet, footbaths not only stimulate local blood circulation by dilating the vessels [13, 14] but also can impact one's overall thermal response [12, 14]. Thus, warm footbaths could theoretically be a potent method for mitigating the disturbances in the thermoregulation of patients with AN. In a previous study with ginger (Zingiber officinale, GI) [15–17] and mustard (Sinapis nigra, MU) footbaths in healthy adults, both thermogenic substances increased subjective warmth at the feet more than a water-only footbath (WA) of the same temperature. For GI, this heightened warmth perception endured for 10 minutes after termination of the footbath [12]. The fact that the active ingredients of GI and MU are able to penetrate skin [18–20] and to activate temperature-sensitive ion channels of the transient receptor potential (TRP) ion channel superfamily [21–23] might explain the potential therapeutic advantage of both thermogenic substances when added to warm footbaths. However, these warming and stimulating effects have so far only been described in healthy adults. It remains unclear whether footbaths containing thermogenic substances have similar effects in individuals with thermoregulatory impairments, such as patients with AN. Our investigation consequently evaluated footbaths containing the medicinal powders of either MU or GI versus WA alone to assess whether they produced varied subjective sensations and objective changes in warmth in specific body regions of interest in patients with AN when compared to healthy controls.

## 2. Materials and Methods

### 2.1. Study Design

This was an explorative randomized vehicle controlled, three-arm trial with a crossover design comparing the thermogenic effects of MU and GI footbaths on psychophysiological parameters in HC and patients with AN. Participants received all three footbath conditions (WA, WA plus MU, and WA plus GI) in a randomized sequence (a total of six possible sequences). The study was approved by the local ethics committee and was registered at the US National Institutes of Health (NCT03519698). The study follows the recommendations of the CONSORT (Consolidated Standards of Reporting trials) statement [24].

### 2.2. Study Population

Patients diagnosed with AN (following the ICD-10 F50.0 criteria) were recruited from the local inpatient clinic, with controls being identified mainly by posting announcements in local school magazines or through direct contact. Eligible participants were female adolescents between 12 and 18 years who assent to participate and whose parents or legal guardians provided written informed consent. Exclusion criteria were pregnancy, infectious diseases (with more than 38°C core body temperature), skin injuries on the lower legs or feet, hypersensitivity to MU or GI products, cardiac arrhythmia, and insufficient knowledge of the German language. In case of HCs, a BMI percentile below 10% was classified as an additional exclusion criterion. Patients with AN were diagnosed by the local inpatient clinic psychiatrist (following the ICD-10 F50.0 criteria) and additionally completed various psychological questionnaires, as did the HCs (see Section 2.6) to assess severity of the eating disorder symptoms. After being enrolled, participants were asked to refrain from consuming nicotine and coffee within three hours before participation in the experiment.

### 2.3. Study Interventions

Each intervention period began with a verbal introduction (2 min), during which participants were instructed to remain in a seated position with their feet and lower legs unclothed throughout the session. A ten-minute equilibration period was next provided to allow the participants' body temperatures to achieve a stable temperature [25, 26]. As suggested in the literature when using human infrared (IR) applications, we endeavored to maintain room temperatures between 18 and 25°C [25, 27]. Footbaths were prepared with 12 liters of water heated to 40.0 ± 1.0°C, placed within plastic tubs, in which water depth was 15 cm. When evaluating MU or GI, 80 grams of prepared powder were added (Sinapis nigrae semen/Zingiberis rhizoma). Following the equilibration period, participants received one of the three footbath conditions according to the randomization schedule. Footbath interventions were interrupted when participants felt uncomfortable or after the maximum limit of 20 minutes to minimize the potential for harm. After removing the feet from the water, participants remained in a seated position for ten additional minutes. We monitored water and room temperature and humidity, as well as the duration of footbath immersion for subsequent analysis. At least 1–3 days were intervened between two consecutive footbath interventions (*M* = 3.82 days, SD = 4.58). All measurements were conducted in the afternoon and early evening between 12:00 and 06:15 pm.

### 2.4. Study Outcomes

All primary and secondary outcome measures were assessed at three specific time points: directly before intervention (baseline or t1), directly after intervention (postimmersion or t2), and ten minutes following the end of the footbaths (follow-up or t3).

The “Herdecke warmth perception questionnaire” (HeWEF) by Edelhäuser et al. [28, 29] was administered to obtain subjective ratings of warmth. The HeWEF is designed to measure perceived sensations of body warmth and warmth distribution for up to 24 body parts (HeWEF state) (Cronbach's *α* = 0.93) as well as general warmth (HeWEF trait). Warmth perception for specific areas are rated on a five-point scale, ranging from 0 (cold) to 4 (hot). We summed up two items of adjacent smaller areas to serve as outcome measures for the feet, hands, and face, with scale scores ranging from 0 to 8. A rating of overall warmth (single item) was obtained using the same 5-point scale, wherein scores ranged from 0 to 4.

Change in actual skin temperature was assessed with a high-definition IR camera (FLIR SC660, FLIR Systems, Wilsonville, Oregon/USA, image resolution 640 × 480 pixels, thermal sensitivity <30 mK). Pictures were taken of the feet (dorsum of feet and toes), hands (back and palms of the hands and fingers), and face (forehead, eye area, inner canthus of the eyes, cheeks, nose, mouth, and chin), while maintaining a distance of two meters between the camera and skin. We obtained precise measures in centigrade using the software ThermaCAM™. Separate mean values were calculated for the feet, hands, and face for analysis.

Participants were interviewed about adverse events (AEs) at t2 and t3.

### 2.5. Primary and Secondary Outcome Measures

Subjective warmth perception at the feet (HeWEF) at t3 was deemed the most important or primary measure of outcome. Secondary outcomes were warmth perceptions at the feet (t1 and t2), face, and hands, subjective overall warmth (t1, t2, and t3), as well as actual skin temperatures (thermography) at the feet, face, and hands (t1, t2, and t3). We gave much consideration to what would best serve as our primary measure of outcome in the planning of this investigation. We initially decided to designate change in overall warmth perception as our primary measure. However, we abandoned this approach as we considered it insufficiently specific. We therefore focused on the main region of interest: the feet. Neither the study design nor the sample considerations were altered by the reordering of our measurement priorities.

### 2.6. Baseline Measurements

We determined the severity of AN with German versions of the validated questionnaires Eating Disorder Inventory-2 (EDI-2) (0.74 ≤ Cronbach's *α* ≤ 0.95) [30] and Eating Disorder Examination Questionnaire (EDE-Q) (0.73 ≤ Cronbach's *α* ≤ 0.86) [31, 32]. The EDI-2 contains 11 subscales (91 total items). Each item is scored on a six-point scale, ranging from 1 (never) to 6 (always). The EDE-Q yields 4 subscales (22 total items) that range between 0 (no day) and 6 (every day). We used the HeWEF trait question “How do you generally feel with respect to body temperature?” to assess baseline differences in participants' subjective warmth perception on a five-point scale, ranging from 1 (cold) to 5 (hot).

### 2.7. Sample Size

We were unable to identify any published studies examining the effects of footbaths with thermogenic substances on psychophysiological parameters in adolescents. We decided not to rely on the study of Vagedes et al. [12] for sample size calculation as this study was conducted with healthy adults. Harju reported age differences in the perceived intensity of warmth at the feet [33]. Thus, warmth perception might differ between adults and adolescents. Furthermore, our main focus is on adolescents with thermoregulatory disturbances and not on healthy individuals. Patients with anorexia nervosa often have internal feelings of being too cold or of being too hot which are not derived from changes in the ambient temperature [1]. The comparison of warmth perception at the feet between healthy adults and diseased adolescents should therefore be made with caution. Thus, parameters needed to estimate sample size were unavailable. By default, a convenience sample of 36 participants was estimated to be sufficient for our purposes. The final number of participants was augmented to 41 due to a better availability of interested adolescents than expected.

### 2.8. Randomization

Participants were randomly allocated to one of the six possible footbath sequences a-f (a = MU-WA-GI, b = MU-GI-WA, c = WA-GI-MU, d = WA-MU-GI, e = GI-MU-WA, and f = GI-WA-MU). We prepared sealed, opaque envelopes containing one of the six possible sequences. At the first testing day, participants drew one of the sealed envelopes in the presence of the nurse research assistant. We recorded the sequence for each participant and provided them a study ID.

### 2.9. Blinding

Data collectors, analysts, and outcome adjudicators were aware of the allocated footbath sequence, so the participants were kept blinded. To prevent potential biased responses due to any visual or olfactory cues, we covered the footbaths with towels during the intervention and used a room spray containing essential oil (between t1 and t2) as masking agents. Participants were asked which odors they perceived predominantly and were permitted to provide multiple response options from the following list: MU, GI, eucalyptus, lavender, citrus, and peppermint. At t2, participants were asked, “which condition did you receive today?” and were permitted to choose between MU, GI, or WA.

### 2.10. Statistical Analysis

All data analyses were conducted with the programming language R (R Core Team, 2018, version 3.5.1) running in RStudio (version 1.1.453). Multiple imputation by chained equations was applied to treat missing values (R package: mice [34]). The significance level was set at *α* = 0.05 (two-tailed). Given our decision to employ a crossover design, we initially assessed for potential asymmetrical sequence effects (due to the interaction between treatment and carryover effects) following the procedure proposed by Wellek and Blettner [35]. We first calculated the (total) sum of all three periods of the initial values (t1) of the primary outcome measure per subject. Failing to find a significant effect in a subsequent one-factorial analysis of variance (ANOVA) with the sequence groups as the factor, we then pooled the groups together for the analysis of intervention effects.

The analysis of our primary outcome measure (HeWEF feet) was performed with the use of a linear mixed effects model (R package: lme4 [36]), with subjects as a random effect and footbath condition (WA, MU, and GI), health status (AN and HC), and time (t1, t2, and t3) as fixed effects. Interaction terms between time and footbath condition as well as between time and health status were also included. Baseline room temperature and humidity were fitted as covariates. Model selection was based on the calculation of 95% confidence intervals (CI), the Akaike and Bayesian information criteria (AIC and BIC), and likelihood ratio statistics.

For the post hoc analysis of warmth perception at the feet at t3, *p* values were estimated from the model for the comparisons between the three footbath conditions (WA vs. MU, WA vs. GI, and MU vs. GI) and between both subgroups (AN vs. HC) using the package lmerTest [37]. A Bonferroni correction was used to adjust for multiple testing within these analyses. Cohen's effect sizes for correlated samples (*d*) were calculated using the model adjusted values. Secondary outcome measures that were not derived from the primary analysis are described descriptively. Mean differences between the footbath conditions as well as between the two subgroups (AN vs. HC) were assessed for all outcome measures at all three time-points with 95% CI and Cohen's *d* effect sizes (R package: effsize [38]). Baseline demographics of the randomization groups are reported descriptively. Welch's unequal variances *t*-tests were used to compare the two study subgroups with respect to the EDI-2, EDE-Q, HeWEF trait subscales, as well as to the baseline warmth perception (HeWEF) and skin temperature (thermography). We analyzed further potential baseline differences with respect to humidity and room and water temperature using two-factorial ANOVAs with footbath condition and health status as the independent variables. Differences in footbath immersion were examined with mixed models allowing for footbath condition, health status, and their interaction as fixed effects and subjects as a random effect. A sensitivity analysis examined the influence of the intention-to-treat approach and the missing value imputation (MI) procedure. We therefore repeated the analysis without MI (ITT-B) and conducted a per-protocol analysis with (PP-A) and without MI (PP-B), comparing the results with the primary analysis (ITT-A). The Cochran–Mantel–Haenszel chi-squared statistic was applied to verify the success of blinding. Potential associations between the footbath conditions MU and GI and subjects' odor perceptions were examined taking the total number of odor perceptions into account as confounder. Data were crosschecked to assess whether they conformed to a normal distribution.

## 3. Results

### 3.1. Study Population

**Figure 1:**
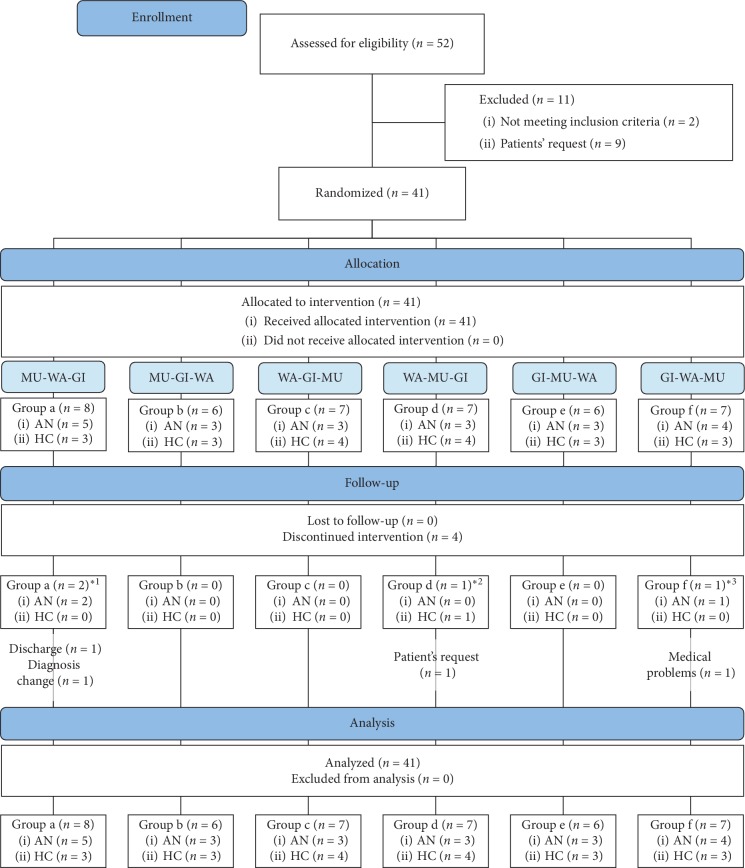
CONSORT flow diagram (^*∗*1^: WA and GI missing; ^*∗*2^: GI missing; ^*∗*3^: WA and MU missing).

The recruitment phase took place between December 2015 and April 2017. Fifty-two female adolescents were screened for eligibility and the 41 adolescents (21 AN and 20 HCs), between 12 and 17 years (*M* = 14.22, SD = 1.54), who met the study criteria were randomly allocated to one of the six sequence groups (a–f) (Figure 1). For the main analysis, the sequence groups were pooled together with regard to the intervention received (based on the crossover design: *n* = 41 for WA, MU, and GI). Four participants discontinued the study protocol (Figure 1). The final cases in each footbath condition were *n* = 38 (AN: *n* = 18 and HC: *n* = 20) for WA, *n* = 40 (AN: *n* = 20 and HC: *n* = 20) for MU, and *n* = 38 (AN: *n* = 19 and HC: *n* = 19) for GI. The mean total time to complete all three interventions was 8.32 days (SD = 7.12, Min = 3.00, and Max = 37.00).

Twenty of the 21 patients with AN were diagnosed with ICD-10 F50.0 (restricting subtype F50.00: *n* = 19; binge eating/purging subtype F50.01: *n* = 1), with the remaining adolescent diagnosed with an atypical AN (ICD-10 F.50.1). Mean duration of illness was 589.57 days (SD = 568.14, Min = 94.00, and Max = 2285.00), and mean hospital stay was 70.33 days (SD = 23.47, Min = 9.00, and Max = 113.00). During this time, 6 patients were given a feeding tube and 13 were medicated with olanzapine (2.5 mg orally). The inpatient treatment program was based on a multimodal treatment approach that was developed in the mid-80s in an German anthroposophic clinic [3, 39]. Significant differences were found between patients with AN and HC with respect to the personal characteristics BMI, EDE-Q, EDI-2, and HeWEF trait subscales, with two exceptions (e.g., the bulimia and maturity fears subscales from EDI-2) (Table 1). At baseline, warmth perception at the feet and hands, subjective overall warmth, as well as skin temperature at the feet, face, and hands were significantly colder in AN compared to HC (Table 2).

### 3.2. Baseline Room and Footbath Conditions

Baseline measures were similar in all three interventions (Table 2). Initial room conditions were a temperature (RT) of 25.04°C (SD = 2.12) and humidity (HM) of 32.15% (SD = 6.29). The mean water temperature (WT) of prepared footbaths was 39.95°C (SD = 0.31). No significant differences occurred with respect to health status (RT: *F* (1, 119) = 3.29, *p*=0.07, HM: *F* (1, 119) < 1, WT: *F* (1, 119) = 1.45, *p*=0.23) or footbath condition (RT: *F* (2, 119) < 1, HM: *F* (2, 119) < 1, WT: *F* (2, 119) = 2.38, *p*=0.10). However, a significant main effect of footbath condition was found for the duration of footbath immersion (*F* (2, 78) = 47.44, *p* < 0.001). Post hoc tests revealed a significantly shorter duration for MU (*M* = 13.02 minutes, SD = 5.26) compared to GI (*M* = 17.78, SD = 3.06) and WA (*M* = 19.41, SD = 1.86) (MU vs. GI: *t*(78) = −6.96, *p* < 0.001, *d* = −1.11; WA vs. GI: *t*(78) = 2.42, *p*=0.05, *d* = 0.65; MU vs. WA: *t*(78) = −9.38, *p* < 0.001, *d* = −1.62). Neither the health status nor the interaction for health status and footbath condition were significant for duration of the footbaths (health status: *F* (1, 39) < 1; interaction term: *F* (2, 78) < 1). Patients with AN received the first footbath measurement on average 13.19 days (SD = 7.72) after hospital admission.

### 3.3. Analysis of Possible Carryover Effects

No difference was found between the total sum scores for warmth perception at the feet for the six different sequence groups at t1 (*F* (5, 35) = 2.04, *p*=0.10). As the possibility for carryover effects was negligible, the groups were pooled together with regard to the intervention received (MU vs. GI vs. WA) (*n* = 41).

### 3.4. Model Selection

We compared four models (wherein participants served as a random effect) in order to obtain the optimal mixed effects analysis for the study's primary outcome measure. In the initial model (model A), a three-way interaction between footbath condition, health status, and time was assumed. The selection of fixed effects for model B was based on the results of an ANOVA of model A incorporating only significant effects (footbath condition, health status, time, interaction between condition and time, and interaction between status and time). The model comparison pointed to a better data approximation through model B (AIC: model A: 1371.0, B: 1369.4; BIC: model A: 1449.2, B: 1424.2; *X*_diff_^2^(6) = 10.39, *p*=0.11). We then extended model B by entering footbath duration, RT and HM as covariates (model C), and compared this model to model B (AIC: 1342.3; BIC: 1408.8; *X*_diff_^2^(3) = 33.05, *p* < 0.001). Based on a 95% CI analysis of model C (footbath duration: −0.06 to 0.04; RT: 0.12 to 0.32; HM: 0.00 to 0.08), we decided to discard the covariate footbath duration while retaining RT and HM as covariates (model D) and compared this model to model C (AIC: 1340.6; BIC: 1403.2; *X*_diff_^2^(1) = 0.29, *p*=0.59). Based on these results, we decided to apply model D for analyzing the primary outcome measure.

### 3.5. Outcomes and Estimations

#### 3.5.1. Primary Outcome Measure

**Figure 2:**
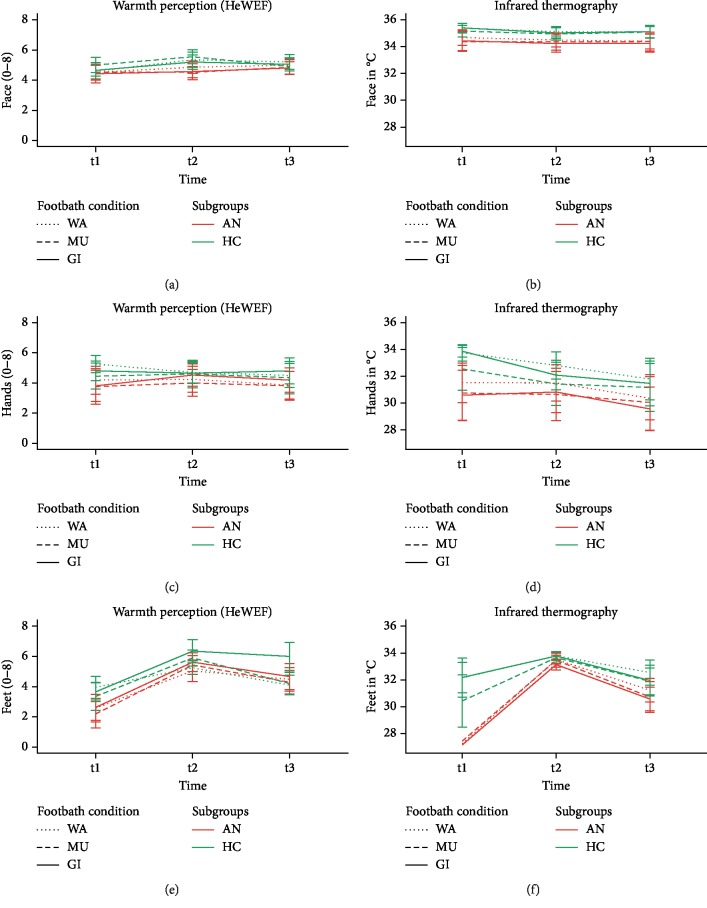
Warmth perception (HeWEF) and infrared thermography at the face, hands, and feet at baseline (t1), postimmersion (t2), and follow-up (t3) (mean values with 95% confidence intervals). Note: WA = water only condition, MU = mustard added to WA, GI = ginger added to water, AN = participants with anorexia nervosa, and HCs = healthy controls.

At t3, warmth perception at the feet was significantly higher after GI compared to WA and MU: GI vs. WA, mean difference −1.02 (95% CI −1.81 to −0.24), *adj.p* < 0.01, *d* = −0.69; GI vs. MU, −1.07 (−1.88 to −0.27), *p* < 0.01, *d* = −0.71. No significant difference was found between WA and MU (mean difference 0.05 (95% CI −0.65 to 0.74), *adj.p*=1.00, *d* = 0.02) (Table 3 and Figure 2). Ratings of warmth perception at the feet at t3 were not reported as different between patients with AN and HC (mean difference −0.29 (95% CI −0.93 to 0.35), *adj.p*=1.00, *d* = −0.07) (Table 4).

#### 3.5.2. Secondary Outcome Measure

At t2, self-perceived warmth was higher after GI compared to WA (mean difference −0.83 (95% CI −1.45 to −0.21), *adj.p*=0.04, *d* = −0.56), with no differences obtained between WA and MU (−0.51 (−1.07 to 0.05), *adj*.*p*=0.59, *d* = −0.36) or between MU and GI (−0.32 (−0.93 to 0.30), *adj.p*=1.00, *d* = −0.21) (Table 3). No differences in warmth perception at the feet at t2 were found between patients with AN and HC (mean difference −0.47 (95% CI −0.96 to 0.02), *adj.p*=1.00, *d* = −0.18) (Table 4).

**Figure 3:**
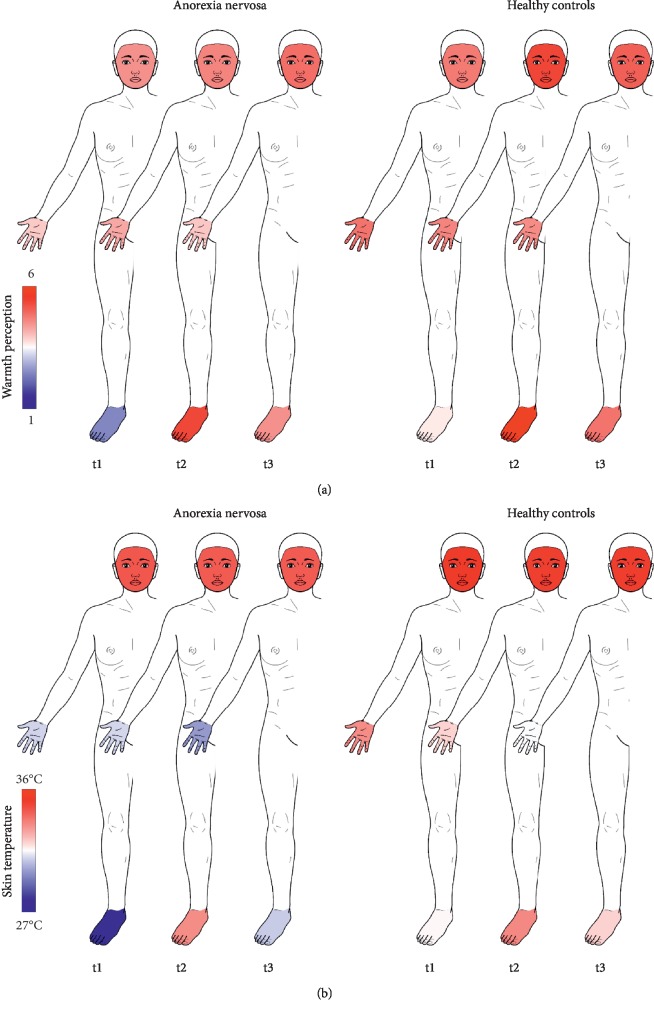
Course of the warmth perception (HeWEF) (a) and skin temperature (infrared thermography) (b) of participants with anorexia nervosa and healthy controls at the face, hands, and feet at baseline (t1), postimmersion (t2), and follow-up (t3). Body template modified from Neubert & Beissner (2019): Hannover Body Template (figshare DOI: https://doi.org/10.6084/m9.figshare.7637387.v5 under CC BY 4.0).

Patients with AN obtained objectively measured (IR) and perceived (HeWEF) temperatures that were colder than those for HC (Table 4 and Figures 2 and 3), with the largest effect sizes occurring for the feet at t1: IR, mean difference −4.31 (95% CI −5.58 to −3.04), *d* = −1.21; HeWEF, −1.19 (−1.83 to −0.55), *d* = −0.66. A principal craniocaudal temperature decrease was noted for the objective skin temperatures (IR) at t1 for patients with AN and HC, but only patients with AN reported differences for the subjective warmth distribution (Table 4). Considering the mean differences of the objective skin temperatures between patients with AN and HC at t1, a craniocaudal gradient was found with the smallest differences at the face (mean difference −0.82 (95% CI −1.24 to −0.40), *d* = −0.69), while the largest differences occurred at the feet (see above for mean difference) (Table 4).

### 3.6. Sensitivity Analysis

Approximately 6% of HeWEF state (5.72%) and IR data (5.83%) were missing and were imputed via MICE. Data showed a normal distribution. We assume a low influence of the missing imputation procedure on the data as we found no differences in the results between ITT-A and ITT-B as well as between PP-A and PP-B. However, when comparing the intention-to-treat approach with the per-protocol analysis, slightly differing results emerged (ITT: significant main effects of interactions between footbath condition and time (*F* (4, 317) = 2.96, *p*=0.02) and between health status and time (*F* (2, 317) = 3.65, *p*=0.03); PP: both interactions were only marginally significant (footbath condition *x* time: *F* (2, 277) = 2.31, *p*=0.06; health status × time: *F* (2, 277) = 3.02, *p*=0.05)).

### 3.7. Success of Blinding

When participants were asked to identify the predominant odor at t1, only 11 were able to name the correct ingredient (MU: *n* = 2, GI: *n* = 9) out of 123 footbaths administered. The most frequent odor perceptions reported were citrus (*n* = 79), lavender (*n* = 20), eucalyptus (*n* = 17), and GI (*n* = 17). We found no significant association between GI footbaths and GI odor perceptions (Mantel–Haenszel *X*^*2*^(1) = 1.73, *p*=0.19] or between MU footbaths and MU odor perceptions (Mantel–Haenszel *X*^*2*^(1) = 0.09, *p*=0.76), indicating the success of blinding at t1. At t2, participants named the correct condition in 78 of 123 possible cases (MU: *n* = 32, WA: *n* = 21, and GI: *n* = 25).

### 3.8. Harms

We recorded 17 AEs (WA: *n* = 0, MU: *n* = 8, GI: *n* = 9) directly after intervention (t2) and 10 AEs (WA: *n* = 2, MU: *n* = 2, GI: *n* = 6) at follow-up (t3). The AEs were burning sensations of skin (t2: MU: *n* = 4, GI: *n* = 5; t3: WA: *n* = 2, GI: *n* = 4), flushing (t2: MU: *n* = 3, GI: *n* = 1; t3: GI *n* = 1), pruritus (t2: GI: *n* = 1; t3: MU: *n* = 1, GI: *n* = 1), fatigue (t2: GI: *n* = 1; t3: MU: *n* = 1), dry skin (t2: WA: *n* = 1), and edema limbs (t2: MU: *n* = 1). None of the AE required medical intervention. Overshooting skin reactions could be avoided through the discontinuation of the footbath interventions when feeling uncomfortable or when reaching the maximum time allowed (20 minutes).

## 4. Discussion

This study is, to the best of our knowledge, the first randomized, controlled trial of footbaths with MU or GI in adolescents with AN and in HC. The major finding is that warmth perception at the feet ten minutes after the end of the footbaths was higher after a footbath with GI than after footbaths with MU or water only of the same temperature. This “longer-lasting” GI effect was seen in healthy adolescents as well as in adolescents with AN. Furthermore, footbath applications enabled both subjective and objective warming of the feet of adolescents with AN.

The feet were the body region with the highest difference in subjective and objective warmth between patients with AN and HC, supporting the importance of focusing on warming the feet in patients with AN. Interestingly, the majority of studies on warming in individuals with AN chiefly rely on thermal vests around the chest, saunas, or high ambient temperatures [9–11]. Footbaths could possibly provide a more practical, economical approach for warming individuals with AN. The physiological effects of footbaths on humans have been attributed to a decrease in sympathetic activity accompanied by an increase in parasympathetic nerve activity [40–42], thus leading to cutaneous vasodilatation and increased peripheral circulation [43, 44]. As demonstrated here, footbaths effect not only local skin temperatures but also overall thermal sensation [12, 45, 46]. Furthermore, our findings suggest that the choice of medicinal powder is important, with GI being shown to have a “longer-lasting” effect on warmth perception [12]. Taylor et al. have shown that the gradual blood vessel dilatation after local heating can last over 25–30 minutes [13]. We speculate that the addition of thermogenic substances may prolong the temperature effects beyond that reported by Taylor et al. The research of Therkleson and Sherwood supports this speculation as they described a subtle warmth after GI compresses that remained over hours [47].

Patients with AN report not only lower core and skin temperatures; they also describe feeling subjectively colder than individuals who are healthy [1, 3, 4]. Interestingly, the craniocaudal gradient in subjective warmth was only seen for patients with AN. This might point to potential differences in thermal sensation between patients (AN) and HC. Sadakata and Yamada found significantly different threshold values for warm sensations at the feet in individuals with cold constitution compared to individuals who were problem free despite equal skin temperatures at the feet at baseline [48]. Our decision to focus on perceived warmth as primary outcome measure was therefore based on two assumptions: (1) thermal sensation varies among individuals; and (2) individuals are mainly be affected by self-perceived changes within the body. Thermal sensation is mediated by temperature-sensitive ion channels, most of which belong to the TRP ion channel superfamily. Activation of these ion channels leads to a depolarization of sensory neurons in the peripheral nervous system, whereby the signal is transmitted to the central nervous system and interpreted as a sensory response [21, 22, 49, 50]. Of note, TRP channels can be activated not only by temperature, voltage, pressure, and osmolarity [22] but also by the binding of chemical ligands such as GI or MU [21–23]. The binding to TRP channels is connected with a release of neuropeptides such as calcitonin gene-related peptides, which in turn are associated with an increased cutaneous blood flow by triggering myocyte relaxation and vasodilatation [51, 52]. The active ingredient of MU, allyl isothiocyanate, activates both TRPV1 (TRP vanilloid 1) and TRPA1 (TRP ankyrin 1) receptors. Shogaols, the active ingredients of GI, activate mainly TRPV1 receptors [21–23]. TRPA1 is a cold receptor, while TRPV1 is classified as a heat receptor [22, 50, 53]. Thus, the coactivation of cold and heat receptors by the active ingredient of MU may explain why the warming effect is not lasting. In contrast, shogaols from GI only activate the heat receptors TRPV1, potentially providing a reason for the “longer-lasting” GI effect. For GI, an increase in adrenal catecholamine secretion similar to capsaicin has also been described, while no effect has been reported for MU [23, 54, 55]. The finding of no differences between patients with AN and HCs can be taken as an indication that somatic thermosensitive afferents in AN react normally to chemical activation of TRPV1 receptors. This is interesting as patients with AN show marked differences in pain perception [56] potentially related to an impaired descending pain modulation [57]. Because TRPV1 is also a nociceptor [22, 49, 58], it may play an important role in this pain modulation process. TRPV1 receptors are activated by endogenous lipid molecules such as anandamide [59], an endocannabinoid, of which patients with AN show elevated blood levels [60]. Anandamide is not only associated with pain pathways in interaction with TRPV1 receptors [59] but is also associated with food craving and food pleasure [61].

The underlying mechanism for the thermoregulatory disturbances in patients with AN with cold blue extremities [1–4] might be a central dysfunction in hypothalamic control centers [1, 4], which in turn is associated with lower thresholds for thermoregulatory sweating and vasodilatation [4]. The drastic reduction in body weight resulting in endocrine changes, loss of corporal insulation, and reduced muscle mass [1, 2], as well as circulatory disturbances (cardiac arrhythmia, hypotension, and bradycardia), result in a reduced blood supply in distal body parts [2]. This cascade of events further contributes to the thermoregulatory impairments. In a functioning thermoregulatory system, thermal homeostasis is preserved by behavioral and autonomic mechanisms [13, 62]. In the event of temperature changes, signals are sent from peripheral (skin) thermoreceptors and internal body temperature sensors to the anterior hypothalamus triggering autonomic thermodefensive responses [13, 63, 64]. Cutaneous vasoconstriction as well as metabolic or shivering thermogenesis resulting in heat conservation are autonomic responses to cold stress, while vasodilatation, sweating, or panting resulting in heat dissipation are the corresponding responses to heat stress [13, 62–64]. Cutaneous blood vessels can be affected by local thermal changes [13]. Local skin heating, for example, leads to a rapid vasodilatation with a large flow increase. However, the central autonomic drive dominates over the external thermal influences. According to Taylor et al., thermal treatments were scarcely able to override vasoconstriction in mildly hypothermic individuals. Accordingly, maximum blood flow in the extremities can only be reached when some levels of hyperthermia is first induced [13]. However, we detected a slight increase in foot skin temperature in patients with AN and HCs. As we did not measure core temperature, we are unable to determine if the changes in skin temperature we noted are associated with changes in the core temperature. The slight decreases in hand and face skin temperature might originate from heat dissipation and evaporation from the skin due to lower room temperatures [14, 43].

As in other studies, the water temperature was not kept constant in all three footbath conditions [12, 41, 42, 65]. Based on in vitro measurements collected in an ongoing study utilizing the same experimental conditions, we hypothesize an average temperature drop of 1.6°C in 20 minutes for all conditions. We thus assume that the water temperature at the end of the present experiment would be approximately 38.4°C. The mean room temperature was slightly higher as suggested by the infrared thermography at the extremities (22–24°C) [25, 27]. However, the majority of investigations describe temperatures ranging from 18–25°C as sufficient to avoid shivering or sweating [25, 27]. Nevertheless, we cannot affirm that all participants felt neutral comfort, and we are aware of individual differences in thermal comfort sensation [63].

Some limitations warrant brief mentioning. First, the room sprays containing essential oils enabled a blinding of the participants at t1. At t2, however, the majority of the participants recognized which kind of footbath condition was applied (63% or 78/123 correct answers). Thus, we were not able to blind the effects of the substances when directly applied to the skin for a majority of participants. Second, while all participants reported being nicotine free at the time of treatment, two participants reported consuming coffee within the defined abstinent period and we decided to include their data. We did not record the consumption of other caffeinated beverages such as energy drinks or tea, as we were unaware of any confirmed potential thermoregulatory effects of caffeine. More to the point, the limited number of studies assessing the effects on peripheral or internal body temperatures reveal conflicting and inconsistent information [66–70]. Third, the only available measure to assess subjective sensations of warmth (HeWEF) has limited psychometric support (articles on validity have yet to be published). Finally, we did not perform an internal validation for the statistical model used for the analysis of our primary outcome measure. Prospective evaluation of our selected model is needed. In future investigations, rigor would be increased by collecting data on the long-term effects of footbaths (e.g., 24–48 hours following the treatment), refining existing measures of subjective warmth, assessing core temperature, and investigating the effects of regular footbath applications (e.g., four times per week for six weeks) on disease etiology and pathogenesis. We limited our focus to female adolescents, as the highest incidence rate for anorexia nervosa is found among adolescent girls between 15 and 19 years [71]. Whether our findings will hold for patients with AN at various ages or for males with AN cannot be determined.

## 5. Conclusions

In conclusion, footbaths with ginger increased perceived warmth at the feet longer than with mustard or warm water only (“longer-lasting” effect) for adolescents with anorexia nervosa as well as for healthy controls. Footbaths could be a useful addition to the multimodal treatment of individuals with AN. If so, care is warranted when selecting the optimal substance to add to the footbaths.

## Figures and Tables

**Table 1:** Personal characteristics (t1) of adolescents with anorexia nervosa (AN, *n* = 21) and healthy controls (HC, *n* = 20).

	AN		HC		*t*	*p*	ES
	M	SD	M	SD			
*Demographic*							
Age (years)	14.14	1.49	14.30	1.63	−0.32	0.75	−0.10
BMI (kg/m^2^)	14.53	0.93	18.93	1.48	−11.32	**<0.001**	−3.58

*Eating Disorder Examination Questionnaire (EDE-Q)*							
Restraint scale	2.78	2.05	0.68	1.06	4.14	**<0.001**	1.27
Eating concern scale	2.82	1.60	0.46	0.81	6.00	**<0.001**	1.85
Weight concern scale	3.12	1.92	0.97	1.22	4.30	**<0.001**	1.33
Shape concern scale	3.71	1.67	1.07	1.15	5.91	**<0.001**	1.83
Total score	3.11	1.67	0.79	0.97	5.45	**<0.001**	1.68

*Eating Disorder Inventory-2 (EDI-2)*							
Drive for thinness	28.29	9.13	15.70	8.29	4.63	**<0.001**	1.44
Bulimia	10.90	3.55	12.55	5.50	−1.13	0.27	−0.36
Body dissatisfaction	36.19	8.57	26.15	9.33	3.58	**<0.001**	1.12
Ineffectiveness	30.71	7.62	22.05	5.04	4.31	**<0.001**	1.33
Perfectionism	18.38	5.24	15.45	3.68	2.08	**0.04**	0.64
Interpersonal distrust	20.57	4.59	16.60	5.00	2.65	**0.01**	0.83
Interoceptive awareness	33.48	9.89	23.00	5.95	4.13	**<0.001**	1.28
Maturity fears	27.76	5.63	24.55	5.77	1.80	0.08	0.56
Asceticism	25.95	5.61	20.60	4.04	3.52	**<0.01**	1.09
Impulse regulation	26.90	6.86	20.55	4.42	3.54	**<0.01**	1.10
Social insecurity	26.62	5.09	20.80	5.78	3.41	**<0.01**	1.07
Total score	284.62	57.93	214.30	44.33	4.38	**<0.001**	1.36

*Herdecke Warmth Perception Questionnaire (HeWEF trait)*							
Trait warmth^a^	2.52	0.75	3.50	0.61	−4.59	**<0.001**	−1.43

Data are means (*M*) and SD of all participants (^a^How do you generally feel with respect to body temperature? (1 = cold, 5 = hot)). Bold indicates a *p* value <0.05.

**Table 2:** Baseline warmth perception and skin temperature in AN (*n* = 21) and HC (*n* = 20) groups.

	Footbath condition			AN vs. HC		
	WA	MU	GI	*t*	*p*	ES
*Warmth perception (as assessed by the Herdecke Warmth Perception Questionnaire)*						

*Feet* ^*a*^						
AN	2.57 ± 2.01	2.19 ± 2.02	2.62 ± 1.88	−3.68	**<0.001**	−*0.66*
HC	3.95 ± 1.54	3.35 ± 1.95	3.65 ± 1.35			
*Face* ^*a*^						
AN	4.52 ± 0.98	4.52 ± 1.17	4.43 ± 1.36	−1.30	0.20	−0.23
HC	4.60 ± 1.19	5.00 ± 1.08	4.65 ± 0.81			
*Hands* ^*a*^						
AN	4.19 ± 2.04	3.76 ± 2.59	3.81 ± 2.29	−2.62	**0.01**	−0.47
HC	5.25 ± 1.21	4.45 ± 1.85	4.80 ± 1.40			
*Overall warmth* ^*b*^						
AN	2.10 ± 0.70	1.90 ± 1.00	1.76 ± 0.94	−2.54	**0.01**	−0.45
HC	2.35 ± 0.49	2.10 ± 0.72	2.35 ± 0.59			

*Skin temperature (as assessed with a high-resolution IR camera in °C)*						

*Feet*						
AN	27.25 ± 3.49	27.44 ± 4.27	27.16 ± 3.63	−6.71	**<0.001**	−**1.21**
HC	32.16 ± 3.12	30.43 ± 4.18	32.18 ± 2.42			
*Face*						
AN	34.68 ± 1.27	34.37 ± 1.55	34.43 ± 1.59	−3.88	**<0.001**	−*0.69*
HC	35.38 ± 0.76	35.16 ± 0.94	35.40 ± 0.71			
*Hands*						
AN	31.52 ± 3.25	30.75 ± 4.54	30.59 ± 4.10	−4.24	**<0.001**	−*0.75*
HC	33.75 ± 1.31	32.55 ± 3.41	33.87 ± 0.91			

Data are means ± SD of all participants (WA = water, MU = mustard, GI = ginger, AN = adolescents with anorexia nervosa, and HC = healthy controls). ^a^Total score between 0 = cold and 8 = hot; ^b^total score between 0 = cold and 4 = hot.

**Table 3:** Mean values for warmth perception and actual skin temperatures at t1, t2, and t3 and mean differences between the footbath conditions (AN and HC data merged together) as a function of time.

	WA (*n* = 41)	MU (*n* = 41)	GI (*n* = 41)	WA vs. MU			WA vs. GI			MU vs. GI		
				Diff1	95% CI	ES	Diff2	95% CI	ES	Diff3	95% CI	ES
Warmth perception (as assessed by the Herdecke Warmth Perception Questionnaire)												

*Feet* ^*a*^												
t1	3.24 ± 1.91	2.76 ± 2.05	3.12 ± 1.71	0.49	(−0.38; 1.36)	0.25	0.12	(−0.67; 0.92)	0.07	−0.37	(−1.19; 0.46)	−0.19
t2	5.15 ± 1.30	5.66 ± 1.26	5.98 ± 1.52	−0.51	(−1.07; 0.05)	−0.40	−**0.83**	(−1.45; −0.21)	−*0.59*	−0.32	(−0.93; 0.30)	−0.23
t3	4.29 ± 1.54	4.24 ± 1.62	5.32 ± 2.02	0.05	(−0.65; 0.74)	0.03	−**1.02**	(−1.81; −0.24)	−*0.57*	−**1.07**	(−1.88; −0.27)	−*0.59*
*Face* ^*a*^												
t1	4.56 ± 1.07	4.76 ± 1.14	4.54 ± 1.12	−0.20	(−0.68; 0.29)	−0.18	0.02	(−0.46; 0.51)	0.02	0.22	(−0.28; 0.72)	0.19
t2	5.10 ± 1.02	5.02 ± 1.04	4.88 ± 1.12	0.07	(−0.38; 0.53)	0.07	0.22	(−0.25; 0.69)	0.20	0.15	(−0.33; 0.62)	0.14
t3	5.10 ± 0.94	4.88 ± 1.08	4.93 ± 0.93	0.22	(−0.23; 0.66)	0.22	0.17	(−0.24; 0.58)	0.18	−0.05	(−0.49; 0.39)	−0.05
*Hands* ^*a*^												
t1	4.71 ± 1.75	4.10 ± 2.26	4.29 ± 1.95	0.61	(−0.28; 1.50)	0.30	0.41	(−0.40; 1.23)	0.22	−0.20	(−1.12; 0.73)	−0.09
t2	4.46 ± 1.72	4.29 ± 1.95	4.59 ± 1.52	0.17	(−0.64; 0.98)	0.09	−0.12	(−0.83; 0.59)	−0.08	−0.29	(−1.06; 0.48)	−0.17
t3	4.17 ± 1.87	4.07 ± 2.18	4.49 ± 1.80	0.10	(−0.80; 0.99)	0.05	−0.32	(−1.12; 0.49)	−0.17	−0.41	(−1.30; 0.47)	−0.21
*Overall warmth* ^*b*^												
t1	2.22 ± 0.61	2.00 ± 0.87	2.05 ± 0.84	0.22	(−0.11; 0.55)	0.29	0.17	(−0.15; 0.49)	0.23	−0.05	(−0.42; 0.33)	−0.06
t2	2.49 ± 0.71	2.49 ± 0.55	2.44 ± 0.59	0.00	(−0.28; 0.28)	0.00	0.05	(−0.24; 0.34)	0.07	0.05	(−0.20; 0.30)	0.09
t3	2.20 ± 0.71	2.32 ± 0.65	2.39 ± 0.67	−0.12	(−0.42; 0.18)	−0.18	−0.20	(−0.50; 0.11)	−0.28	−0.07	(−0.36; 0.22)	−0.11

*Skin temperature (as assessed with a high-resolution IR camera in °C)*												

*Feet*												
t1	29.64 ± 4.11	28.90 ± 4.44	29.61 ± 3.98	0.74	(−1.14; 2.62)	0.17	0.04	(−1.74; 1.82)	0.01	−0.70	(−2.56; 1.15)	−0.17
t2	33.65 ± 0.71	33.56 ± 1.02	33.45 ± 0.86	0.10	(−0.29; 0.49)	0.11	0.20	(−0.15; 0.55)	0.26	0.10	(−0.31; 0.52)	0.11
t3	31.87 ± 2.06	31.30 ± 2.39	31.25 ± 2.28	0.58	(−0.40; 1.56)	0.26	0.63	(−0.33; 1.58)	0.29	0.05	(−0.98; 1.08)	0.02
*Face*												
t1	35.02 ± 1.10	34.75 ± 1.34	34.90 ± 1.32	0.27	(−0.27; 0.81)	0.22	0.11	(−0.42; 0.65)	0.09	−0.15	(−0.74; 0.43)	−0.12
t2	34.80 ± 1.04	34.65 ± 1.22	34.62 ± 1.24	0.15	(−0.35; 0.65)	0.13	0.18	(−0.32; 0.68)	0.16	0.03	(−0.51; 0.57)	0.02
t3	34.75 ± 1.17	34.72 ± 1.29	34.68 ± 1.32	0.02	(−0.52; 0.57)	0.02	0.07	(−0.48; 0.62)	0.06	0.05	(−0.53; 0.62)	0.04
*Hands*												
t1	32.61 ± 2.71	31.62 ± 4.08	32.19 ± 3.40	0.98	(−0.54; 2.51)	0.28	0.42	(−0.93; 1.77)	0.14	−0.56	(−2.21; 1.09)	−0.15
t2	32.14 ± 2.66	31.03 ± 3.88	31.44 ± 2.96	1.11	(−0.35; 2.58)	0.33	0.70	(−0.53; 1.94)	0.25	−0.41	(−1.93; 1.11)	−0.12
t3	31.06 ± 3.47	30.59 ± 4.19	30.50 ± 3.65	0.47	(−1.22; 2.16)	0.12	0.56	(−1.00; 2.13)	0.16	0.10	(−1.63; 1.82)	0.02

Data are means ± SD (WA = water, MU = mustard, GI = ginger, Diff1 = mean difference of WA minus MU, Diff2 = mean difference of WA minus GI, Diff3 = mean difference of MU minus GI, CI = confidence interval, and ES = Cohen's *d* effect size). ^a^Total score between 0 = cold and 8 = hot; ^b^total score between 0 = cold and 4 = hot). Bold indicates confidence intervals that do not contain zero.

**Table 4:** Mean values for warmth perception and actual skin temperature at t1, t2, and t3 and mean differences between the study groups (WA, MU, and GI data merged together) as a function of time.

	AN (*n* = 21)	HC (*n* = 20)	Diff	95% CI	ES
*Warmth perception (as assessed by the Herdecke Warmth Perception Questionnaire)*					

*Feet^a^*					
t1	2.46 (1.95)	3.65 (1.62)	−**1.19**	(−1.83; −0.55)	−*0.66*
t2	5.37 (1.43)	5.83 (1.33)	−0.47	(−0.96; 0.02)	−0.34
t3	4.48 (1.73)	4.77 (1.86)	−0.29	(−0.93; 0.35)	−0.16
*Face* ^*a*^					
t1	4.49 (1.16)	4.75 (1.04)	−0.26	(−0.65; 0.13)	−0.23
t2	4.65 (0.97)	5.37 (1.02)	−**0.72**	(−1.07; −0.36)	−*0.72*
t3	4.89 (0.94)	5.05 (1.03)	−0.16	(−0.51; 0.19)	−0.16
*Hands* ^*a*^					
t1	3.92 (2.29)	4.83 (1.52)	−**0.91**	(−1.60; −0.22)	−0.47
t2	4.25 (1.80)	4.65 (1.63)	−0.40	(−1.01; 0.22)	−0.23
t3	3.95 (1.95)	4.55 (1.93)	−0.60	(−1.29; 0.09)	−0.31
*Overall warmth* ^*b*^					
t1	1.92 (0.89)	2.27 (0.61)	−**0.35**	(−0.62; −0.08)	−0.45
t2	2.38 (0.71)	2.57 (0.50)	−0.19	(−0.40; 0.03)	−0.30
t3	2.19 (0.64)	2.42 (0.70)	−0.23	(−0.47; 0.01)	−0.34

*Skin temperature (as assessed with a high-resolution IR camera in °C)*					

*Feet*					
t1	27.28 (3.75)	31.59 (3.37)	−**4.31**	(−5.58; −3.04)	−**1.21**
t2	33.37 (0.94)	33.74 (0.76)	−**0.37**	(−0.67; −0.06)	−0.43
t3	30.84 (2.14)	32.13 (2.19)	−**1.29**	(−2.06; −0.52)	−*0.60*
*Face*					
t1	34.49 (1.46)	35.31 (0.80)	−**0.82**	(−1.24; −0.40)	−*0.69*
t2	34.36 (1.31)	35.03 (0.89)	−**0.66**	(−1.06; −0.27)	−*0.59*
t3	34.35 (1.39)	35.10 (0.96)	−**0.76**	(−1.18; −0.33)	−*0.63*
*Hands*					
t1	30.95 (3.96)	33.39 (2.22)	−**2.43**	(−3.57; −1.29)	*-0.75*
t2	30.99 (3.55)	32.11 (2.73)	−1.12	(−2.24; 0.01)	−0.35
t3	29.99 (3.85)	31.48 (3.53)	−**1.49**	(−2.80; −0.17)	−0.40

Data are means ± SD (WA = water, MU = mustard, GI = ginger, Diff = mean difference of AN minus HC, CI = confidence interval, and ES = Cohen's *d* effect size). ^a^Total score between 0 = cold and 8 = hot; ^b^total score between 0 = cold and 4 = hot. Bold indicates confidence intervals that do not contain zero.

## Data Availability

The data used to support the findings of this study are available from the corresponding author upon request.
